# First report of the chromosomal integration of carbapenemase gene *bla*_IMP-19_ in *Acinetobacter baumannii* AB322: the legacy of integron in phage-plasmid?

**DOI:** 10.1128/spectrum.00382-24

**Published:** 2024-04-23

**Authors:** Yung-Luen Shih, Carl Jay Ballena Bregente, Pek Kee Chen, Tran Thi Dieu Thuy, Yu-Chen Chen, Han-Yueh Kuo, Hsu-Feng Lu, Cheng-Yen Kao

**Affiliations:** 1 School of Medicine, College of Medicine, Fu-Jen Catholic University, New Taipei, Taiwan; 2 Department of Pathology and Laboratory Medicine, Shin Kong Wu Ho-Su Memorial Hospital, Taipei, Taiwan; 3 Institute of Microbiology and Immunology, College of Life Sciences, National Yang Ming Chiao Tung University, Taipei, Taiwan; 4 College of Medical Technology, Southwestern University PHINMA, Cebu, Philippines; 5 Department of Medical Laboratory Science and Biotechnology, China Medical University, Taichung, Taiwan; 6 National Taiwan University Hospital Hsin-Chu Branch, Hsin-Chu, Taiwan; 7 National Taiwan University Hospital, National Taiwan University College of Medicine, Taipei, Taiwan; 8 Department of Laboratory Medicine, China Medical University Hospital, Taichung, Taiwan; 9 Department of Medical Laboratory Science and Biotechnology, Asia University, Taichung City, Taiwan; 10 Health Innovation Center, National Yang Ming Chiao Tung University, Taipei, Taiwan; 11 Microbiota Research Center, National Yang Ming Chiao Tung University, Taipei, Taiwan; Universidad de Buenos Aires, Buenos Aires, Argentina

**Keywords:** *bla*_IMP-19_, chromosomal encoded carbapenemase, integron, phage, whole-genome sequencing

## Abstract

Integration of carbapenemase gene *bla*_IMP_ into the chromosome of carbapenem-resistant *Acinetobacter baumannii* (CRAB) has not been reported. The aim of this study was to explore the genomic characteristics of CRAB AB322 isolated from a Taiwanese patient diagnosed with bacteremia in 2011, whose chromosome harbors *bla*_IMP-19_. Disk diffusion and broth microdilution were employed to analyze the antimicrobial susceptibility of AB322 to 14 antimicrobials. Nanopore whole-genome sequencing platform was utilized for AB322 genome sequencing, and conjugation was further performed to investigate the transferability of *bla*_IMP-19_ to amikacin-resistant *A. baumannii* 218 (AB218) and *Acinetobacter nosocomialis* 254 (AN254). The results showed that AB322 was classified as multidrug-resistant *A. baumannii* but remained susceptible to ampicillin/sulbactam, colistin, and tigecycline. Whole-genome sequencing revealed the AB322 genome, consisting of a 4,098,985-bp chromosome, a 71,590-bp conjugative plasmid named pAB322-1, and an 8,726-bp plasmid named pAB322-2. Multilocus sequence typing analysis indicated that AB322 belonged to sequence type 1. AB322 chromosome harbored numerous acquired antimicrobial resistance genes, including *aph(3′)-Ia*, *aadA1b*, *aadA1*, *aac(6′)-Ib3*, *aac (3)-Ia*, *bla*_ADC-25_, *bla*_OXA-69_, *bla*_IMP-19_, *catA1*, *sul1*, and *tet(A*), conferring resistance to β-lactams, aminoglycosides, chloramphenicol, sulfamethoxazole, and tetracyclines. Moreover, *bla*_IMP-19_ was identified to be situated within class 1 integron In240 and an incomplete PHAGE_Salmon_SJ46_NC_031129 on AB322 chromosome. However, conjugation experiments revealed that *bla*_IMP-19_ could not be transferred to AB218 and AN254 in our testing conditions. In conclusion, we first report the presence of chromosomal-integrated *bla*_IMP-19_ in CRAB, possibly mediated by integron. The future dissemination of *bla*_IMP-19_ among different species, leading to carbapenem resistance dissemination, requires close monitoring.

**IMPORTANCE:**

The horizontal transfer of antimicrobial-resistant genes is crucial for the dissemination of resistance, especially as *Acinetobacter baumannii* has emerged as a clinically significant pathogen. However, in this study, we first report the integration of the *bla*_IMP-19_ gene into the chromosome of *A. baumannii*, and such horizontal transfer may be associated with integron-phage elements. Additionally, it is possible that these DNA fragments carrying antimicrobial-resistant genes could further spread to other pathogens by moving horizontally onto conjugative plasmids.

## OBSERVATION

Infections caused by *Acinetobacter baumannii* are frequently severe and challenging to manage due to elevated antimicrobial resistance rates and heightened virulence. Carbapenems, commonly employed to combat life-threatening infections caused by multidrug-resistant (MDR) *A. baumannii*, face a growing challenge with increasing proportions of carbapenem-resistant *A. baumannii* (CRAB). Apart from porin dysfunction and efflux pump overexpression (1, 2), carbapenemase genes present on the bacterial chromosome and plasmids are frequently linked to mobile genetic elements [including insertion sequences (ISs, part of transposon) and integrons], contributing to carbapenem hydrolysis and resistance (3, 4). *bla*_NDM-1_ is the most prevalent carbapenem resistance gene identified in CRAB, followed by oxacillinase genes (OXAs, class D β-lactamases) and *bla*_IMP-1_ (4). Furthermore, these carbapenemase genes in CRAB are primarily disseminated through plasmid-mediation with prevalent genes being *bla*_OXA-24_, *bla*_OXA-58_, *bla*_NDM_, and *bla*_GES_. In contrast, *bla*_NDM_ dominates in the occurrence of carbapenemase genes on CRAB chromosomes (4).

In our previous surveillance, we isolated an MDR-CRAB isolate, AB322, carrying *bla*_IMP-19_, from a patient with bacteremia in Taiwan in 2001 (5). AB322 was resistant to various antimicrobials, including amikacin, cefepime, ceftazidime, ciprofloxacin, gentamicin, imipenem, levofloxacin, meropenem, piperacillin/tazobactam, sulfamethoxazole/trimethoprim, and tetracycline. Moreover, AB322 exhibited high resistance to carbapenems: the MIC value for imipenem is >256 µg/mL, and the MIC value for meropenem is 64 µg/mL. However, AB322 was susceptible to ampicillin/sulbactam, colistin, and tigecycline.

The complete genome sequence of AB322 was determined through Nanopore whole-genome sequencing platform (6, 7) to investigate the features of AB322 genome. Genome sequencing results unveiled the AB322 genome, comprising a 4,098,985-bp chromosome, a 71,590-bp plasmid named pAB322-1, and an 8,726-bp plasmid named pAB322-2. The guanine-cytosine (GC) content of the AB322 chromosome was 39.3%, while that of pAB322-1 and pAB322-2 was 33.6% and 34.4%, respectively. Results from NCBI PGAP annotation revealed that the AB322 chromosome harbored 3,262 coding sequences and 96 RNA genes. Plasmids pAB322-1 and pAB322-2 contained 100 and 11 protein-coding sequences, respectively. The multilocus sequence typing (MLST) analysis (8) indicated that AB322 belonged to sequence type 1 (ST1). Numerous acquired antimicrobial resistance genes (ARGs) were found in the AB322 chromosome, including *aph(3′)-Ia*, *aadA1b*, *aadA1*, *aac(6′)-Ib3*, *aac (3)-Ia*, *bla*_ADC-25_, *bla*_OXA-69_, *bla*_IMP-19_, *catA1*, *sul1*, and *tet(A*), contributing to resistance to β-lactams, aminoglycosides, chloramphenicol, sulfamethoxazole, and tetracyclines (Table S1; Fig. S1).

*bla*_IMP-19_ was initially identified in *Enterobacter cloacae* and *Pseudomonas aeruginosa* from Japan. Subsequently, it was found in an *Aeromonas caviae* isolate in France (9) and, more recently, in *Achromobacter xylosoxidans* and *Acinetobacter* spp. isolates from Japan (10, 11). In a 2011 study by Yamamoto et al., it was noted that *bla*_IMP-19_ could be transmitted via plasmids between *A. baumannii*, *Acinetobacter junii*, and genospecies 3 (11). Subsequently, they reported the widespread distribution of *Acinetobacter pittii* ST119 harboring *bla*_IMP-19_ throughout the Kyoto-Shiga region in Japan (12). However, existing literature has primarily emphasized the plasmid-mediated dissemination of *bla*_IMP-19_. In this study, we report the first occurrence of *bla*_IMP-19_ in *A. baumannii* and discover the integration of *bla*_IMP-19_ into the chromosome.

**Fig 1:**
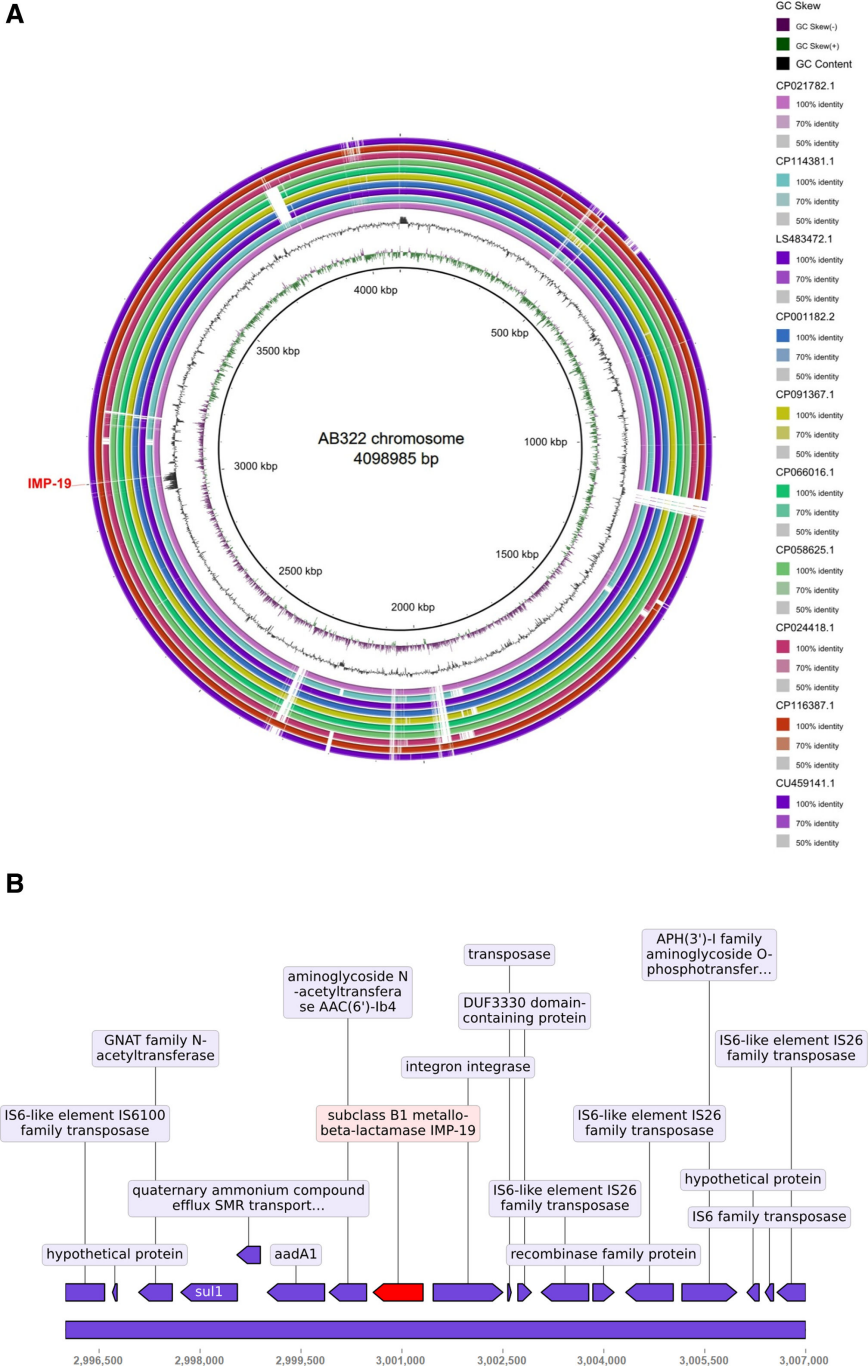
(**A**) Comparative genomics of 10 chromosomes closely related to AB322. BRIG diagram illustrates homologous chromosome segments with the AB322 genome as a reference, highlighting the presence of *bla*_IMP-19_. (**B**) Detailed depiction of the neighboring genomic region from 2,996,000 to 3,007,000 of *bla*_IMP-19_ within the AB322 chromosome was performed using the DNA features viewer in Python libraries. The *bla*_IMP-19_ gene (from 3,000,573 to 3,001,313) was annotated, and its representation was visually highlighted with a red coloration.

**Fig 2:**
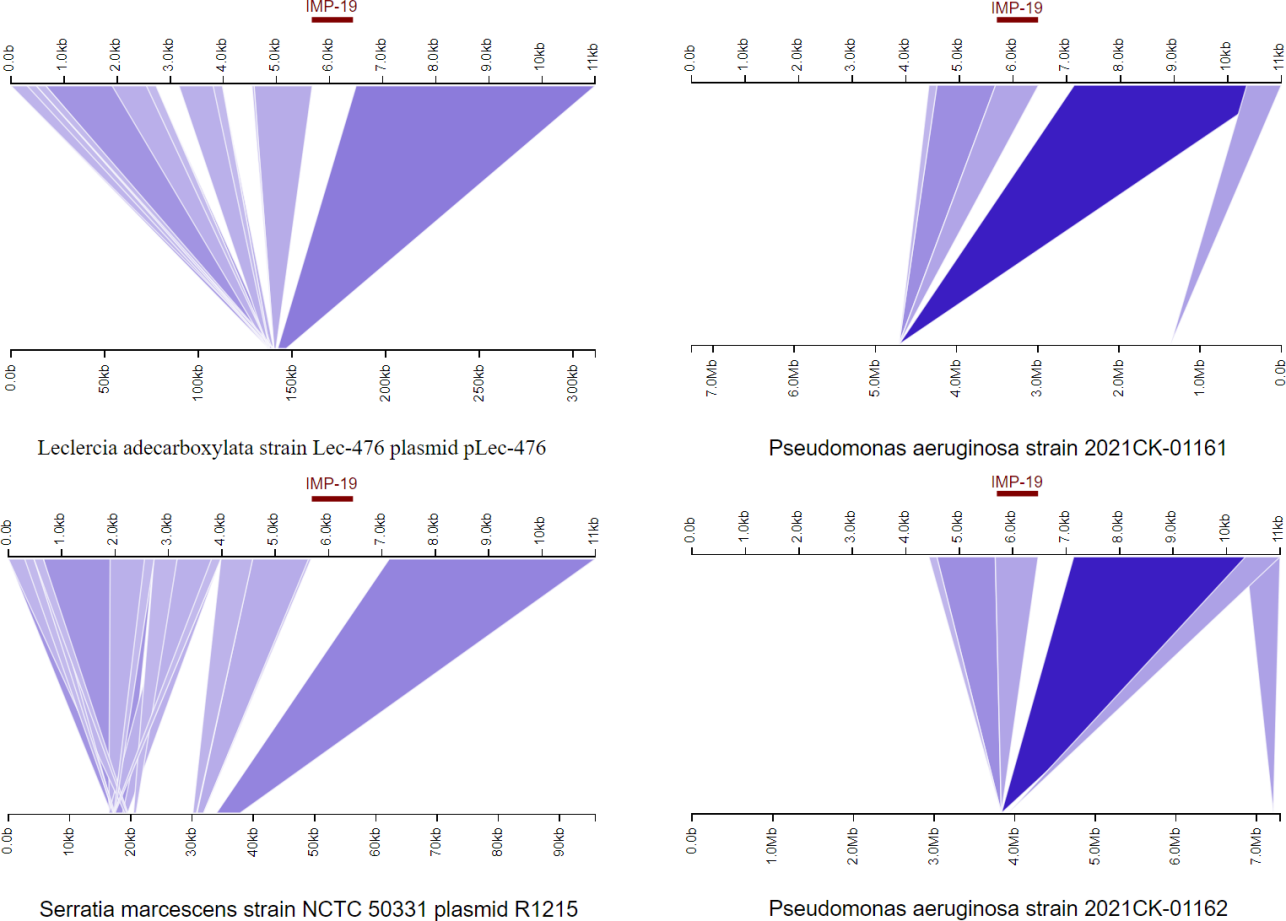
Analyzing the structural comparison diagram using Kablammo. The genomic structure neighboring AB322 *bla*_IMP-19_ (region from 2,996,000 to 3,007,000) was compared with two plasmids (pLec-476 and R1215) and two chromosomal sequences (*P. aeruginosa* 2021CK-00161 and *P. aeruginosa* 2021CK-00162).

Ten chromosomes exhibiting the highest maximum score and substantial similarity (exceeding 95% query coverage and 99.95% identity) to the AB322 chromosome sequence were chosen for further analysis. BLAST Ring Image Generator (BRIG) (13) was employed to generate visual representations illustrating comparisons among the 11 chromosomes. Nevertheless, significant distinctions exist between AB322 and other chromosomes (depicted by white gaps in Fig. 1A), particularly in the exclusive presence of *bla*_IMP-19_ in AB322, which may be caused by mobile genetic element integration. The characterization of mobile genetic elements (integrons and ISs) showed that AB322 chromosome had 2 integrons and 16 transposons (Tables S2 and S3). Importantly, *bla*_IMP19_ was included in integron In240 and surrounded by IS*6*-like elements, IS*26* and IS*6100* (Fig. 1B; Table S3). We conducted a BLAST comparison using the *bla*_IMP19_-including segment (11,000 bp) and identified a total of 60 sequences with coverage ranging from 60% to 100%, consisting of 41 plasmid sequences and 19 chromosome sequences (identity range from 97.76% to 99.92%). Notably, only *P. aeruginosa* strain 2021CK-01104 chromosome (CP137913.1), *P. aeruginosa* strain 2021CK-01161 chromosome (CP124626.1), and *P. aeruginosa* strain 2021CK-01162 chromosome (CP124632.1) were found to carry a *bla*_IMP-18_ gene within the similar 11,000-bp segment. We further selected two plasmids (pLec-476: accession number: KY320277, coverage: 84%, identity: 99.73%; R1215: accession number:NZ_KU315015, coverage: 84%, identity: 99.69%), as well as two chromosomal sequences carrying the *bla*_IMP-18_ gene (*P. aeruginosa* 2021CK-00161: coverage: 63%, identity: 99.92%; *P. aeruginosa* 2021CK-00162: coverage: 63%, identity: 99.92%), for comparison with the 11,000 bp sequence of AB322 neighboring genomic region of *bla*_IMP-19_ by using Kablammo (14) (Fig. 2). The results indicate that the plasmids contain highly similar sequences to the AB322 *bla*_IMP-19_-neighboring segment, potentially serving as reservoirs for ARGs, further contributing to their future dissemination.

We further explored the integration of the *bla*_IMP_ gene into the chromosomes of strains using the NCBI nucleotide database for BLAST analysis. The results revealed that, in addition to AB322, 55 other strains had the *bla*_IMP_ integrated into their chromosomes (Tables S4 and S5). We found two strains of *P. aeruginosa* with duplicated integrons in their chromosomes: strain HPA0875 harbored In887, carrying *aadA1*, *bla*_OXA-1_, and *bla*_IMP-6_, while strain PA99 carried In994, containing *bla*_IMP-1_(Table S5). On the chromosome of *K. pneumoniae* CPO109, there are two distinct integrons. In240 carried *bla*_IMP-4_ and *catB3*, while In498 harbors *bla*_IMP-4_, *catB3*, and *emrB*. Meanwhile, three strains of *P. aeruginosa*, HPA0044, HPA0384, and HPA1406, each carry two *bla*_IMP_-integrons on their chromosomes, In498 and In887, and both integrons contain ARGs *aadA1*, *bla*_OXA-1_, and *bla*_IMP-6_. However, *Morganella morganii* N18-00103, *Proteus mirabilis* N18-00201, *Providencia stuartii* 2021CK-01196, and *P. stuartii* 2021CK-01296 all carried the *bla*_IMP-27_ gene on their chromosomes, but these genes were not located within integrons. In summary, In498 is the most common *bla*_IMP_-integron, not only in *P. aeruginosa*. Additionally, *bla*_IMP-79_ is the most common chromosomally encoded *bla*_IMP_ gene (Table S4).

Krahn et al. previously reported that the intraspecies transfer of the chromosomal *A. baumannii bla*_NDM-1_ gene might occur through phage-mediated transmission (15). Moreover, Pfeifer et al. showed that phage-plasmids obtained from clinical environments have substantiated their ability to infect susceptible strains, leading to the development of antimicrobial resistance (16). Our genome sequence analysis revealed five intact, one questionable, and three incomplete prophages in AB322. The *bla*_IMP-19_ gene was located in region 7 (incomplete *Salmonella* phage SJ46) (Table S6; Fig. S2). We analyzed the distribution of phage SJ46 (accession number NC_031129.1) among different bacteria using the NCBI database. Among the 90 identified sequences of SJ46, 88 were found to belong to plasmids. SJ46 was only detected in two chromosomes (*Escherichia coli* strains ST2747 and RM-096-CS), supporting the possibility of movement between chromosomes and plasmids of SJ46.

Conjugation is another pathway for genetic material transfer, and pAB322-1 possesses a type F conjugation system. Therefore, the liquid mating-out assay (5) was performed to determine the transferability of *bla*_IMP-19_ gene from AB322 isolates to amikacin-resistant *A. baumannii* 218 (AB218) and *Acinetobacter nosocomialis* 254 (AN254). However, we were unable to obtain transconjugants in our tested conditions.

We report for the first time the presence of *bla*_IMP-19_ in *A. baumannii* chromosome, and the *bla*_IMP-19_ gene may integrate into the chromosome via integron-plasmid-phage mediation, eventually evolving into an incomplete phage. The concern lies in disseminating ARGs facilitated by phage-plasmids, as it circumvents the need for cell-to-cell contact essential in traditional plasmid transfer by conjugation. However, the mechanism of integrated carbapenemase in pathogens still needs further investigation. Moreover, it is urgent to monitor the spread of *A. baumannii* ST1 isolates carrying *bla*_IMP-19_ on chromosomes in the environment and their evolution towards co-occurring with multiple ARGs.

## Data Availability

Whole-genome sequencing data are available in the NCBI database under BioProject accession PRJNA938819 and BioSample accession SAMN33446574. The complete genome sequences of the *A. baumannii* isolate AB322 have been deposited in GenBank under the accession numbers CP119232 (AB322 chromosome), CP119235 (pAB322-1), and CP119234 (pAB322-2).

## SUPPLEMENTAL MATERIAL

Supplemental material spectrum.00382-24-s0001.doc Tables S1-S6; Fig. S1 and S2.

ASM does not own the copyrights to Supplemental Material that may be linked to, or accessed through, an article. The authors have granted ASM a non-exclusive, world-wide license to publish the Supplemental Material files. Please contact the corresponding author directly for reuse.
