# Supplementary material for: First report of the chromosomal integration of carbapenemase gene blaIMP-19 in Acinetobacter baumannii AB322: the legacy of integron in phage-plasmid?
Source: Microbiol Spectr. 2024 Apr 23;12(6):e00382-24. doi: 10.1128/spectrum.00382-24 (PMC11237583; doi:10.1128/spectrum.00382-24)
Supplement: Supplemental material — Tables S1-S6; Fig. S1 and S2. [file spectrum.00382-24-s0001.doc]

**Table S1. The presence of acquired antimicrobial resistance genes on AB322 chromosome and pAB322-1.**

| **Antimicrobial resistance genes** | **Position** | **Identity (%)** | **Characteristics (antimicrobial resistance)** |
| --- | --- | --- | --- |
| **Chromosome** |  |  |  |
| *aph(3')-Ia* | 2973491-2974304  3005161-3005974 | 99.75 | kanamycin, neomycin, lividomycin, paromomycin, ribostamycin |
| *aadA1b* | 2999059-2999850 | 99.87 | streptomycin, spectinomycin |
| *aadA1* | 2967324-2968115 | 99.75 | streptomycin, spectinomycin |
| *aac(6')-Ib3* | 2999920-3000474 | 99.64 | tobramycin, amikacin |
| *aac(3)-Ia* | 2969159-2969692 | 99.06 | gentamicin, astromicin, fortimicin |
| *bla*_ADC-25_ | 368022-369173 | 96.07 | beta-lactam |
| *bla*_OXA-69_ | 1071127-1071951 | 100.00 | amoxicillin, ampicillin |
| *bla*_IMP-19_ | 3000573-3001313 | 100.00 | amoxicillin, amoxicillin+clavulanic acid, ampicillin, ampicillin+clavulanic acid, cefepime, cefixime, cefotaxime, cefoxitin, ceftazidime, ertapenem, imipenem, meropenem, piperacillin, piperacillin+tazobactam |
| *catA1* | 2984463-2985121 | 99.70 | chloramphenicol |
| *sul1* | 2997715-2998554  2965980-2966819 | 100.00 | sulfamethoxazole |
| *tet(A)* | 2988966-2990162 | 99.67 | tetracycline, doxycycline |
| **pAB322-1** |  |  |  |
| *aph(3')-VIa* | 39099-39877 | 99.87 | gentamicin, amikacin, kanamycin, neomycin, paromomycin, ribostamycin, butirosin |

The ResFinder 4.4.2 server (http://www.genomicepidemiology.org/) was employed to identify antimicrobial resistance genes.

No acquired resistance genes were identified in **pAB322-2.**

****Table S2. Transposons identified in AB322 chromosome and pAB322-1.****

| **Transposon type (Accession number)** | **Identity (%)** | **Coverage (%)** | **Length (bp)** | **Query Start** | **Query End** | **Subject start** | **Subject end** | **E-value** | **Bit-score** |
| --- | --- | --- | --- | --- | --- | --- | --- | --- | --- |
| **Chromosome** |  |  |  |  |  |  |  |  |  |
| Tn6168 (CP001182) | 98.25 | 78.58 | 4337 | 366043 | 370360 | 4335 | 1 | 0.0 | 7470 |
| Tn6018-L (CP001182) | 99.67 | 100.0 | 3376 | 2959888 | 2963260 | 3376 | 1 | 0.0 | 6033 |
| Tn21 (CP001182) | 100.0 | 100.0 | 447 | 2970901 | 2971347 | 447 | 1 | 0.0 | 807 |
| Tn6292 | 99.88 | 64.93 | 822 | 2971347 | 2972168 | 1266 | 445 | 0.0 | 1478 |
| Tn6020 (CP001182) | 99.87 | 100.0 | 3070 | 2972604 | 2975671 | 3070 | 1 | 0.0 | 5515 |
| Tn4352 (HQ840942) | 98.2 | 64.3 | 1724 | 2972605 | 2974305 | 1 | 1724 | 0.0 | 2982 |
| Tn6292 | 99.52 | 65.09 | 825 | 2974852 | 2975676 | 1265 | 442 | 0.0 | 1467 |
| Tn6292 | 99.64 | 65.56 | 830 | 2979228 | 2980057 | 436 | 1265 | 0.0 | 1483 |
| Tn801 (AF080442) | 99.88 | 69.81 | 3455 | 2980055 | 2983508 | 3455 | 1 | 0.0 | 6210 |
| Tn9-like (HQ840942) | 99.91 | 98.41 | 2227 | 2983508 | 2985733 | 37 | 2263 | 0.0 | 4004 |
| Tn5073 (AF461013) | 98.87 | 100.0 | 4256 | 2991295 | 2995503 | 1 | 4256 | 0.0 | 7488 |
| Tn21 (CP001182) | 100.0 | 100.0 | 447 | 3002572 | 3003018 | 447 | 1 | 0.0 | 807 |
| Tn6292 | 99.76 | 64.93 | 822 | 3003018 | 3003838 | 1266 | 445 | 0.0 | 1471 |
| Tn6020(CP001182) | 99.87 | 100.0 | 3070 | 3004274 | 3007341 | 3070 | 1 | 0.0 | 5515 |
| Tn4352 (HQ840942) | 98.2 | 64.3 | 1724 | 3004275 | 3005975 | 1 | 1724 | 0.0 | 2982 |
| Tn6292 | 99.64 | 64.85 | 821 | 3006522 | 3007342 | 1265 | 445 | 0.0 | 1467 |
| **pAB322-1** |  |  |  |  |  |  |  |  |  |
| TnaphA6 (KU549175) | 99.93 | 93.39 | 2870 | 37987 | 40855 | 3072 | 203 | 0.0 | 5164 |

BacAnt (1) was used to identify transposons.

**Table S3. Two integrons were identified in AB322 chromosome.**

| **ID_integron** | **Position start** | **Position end** | **Strand** | **E-value** | **Annotation** | **Considered_topology** | **Most like integron** |
| --- | --- | --- | --- | --- | --- | --- | --- |
| integron_01 | 2966813 | 2967160 | -1 | 8.7e-29 | emrE | circ | In240|EF368053;  identity:92.85;  coverage:60.36 |
| integron_01 | 2967263 | 2967322 | -1 | 1e-07 | attC | circ |
| integron_01 | 2967324 | 2968103 | -1 | 1.3999999999999997e-165 | ANT3 | circ |
| integron_01 | 2968517 | 2968587 | -1 | 0.0067 | attC | circ |
| integron_01 | 2968528 | 2969040 | -1 | NA | protein | circ |
| integron_01 | 2969056 | 2969164 | -1 | 1.1e-05 | attC | circ |
| integron_01 | 2969159 | 2969692 | -1 | 1.1999999999999997e-107 | AAC3-I | circ |
| integron_01 | 2969794 | 2970828 | 1 | 4.2e-23 | intI | circ |
| integron_02 | 2998548 | 2998895 | -1 | 8.7e-29 | emrE | circ | In240|EF368053;  identity:94.78;  coverage:89.66 |
| integron_02 | 2998998 | 2999057 | -1 | 1.8e-06 | attC | circ |
| integron_02 | 2999059 | 2999838 | -1 | 9.999999999999998e-165 | ANT3 | circ |
| integron_02 | 2999854 | 2999925 | -1 | 0.00059 | attC | circ |
| integron_02 | 2999920 | 3000438 | -1 | 4.899999999999999e-125 | AAC6-Ib | circ |
| integron_02 | 3000493 | 3000570 | -1 | 0.004 | attC | circ |
| integron_02 | 3000573 | 3001313 | -1 | 3.899999999999999e-163 | IMP | circ |
| integron_02 | 3001465 | 3002499 | 1 | 4.2e-23 | intI | circ |

BacAnt (1) was used to identify integrons and their composition.

****Table S4. Correlation between integron types and various *bla*_IMP_ variants in the chromosomes of diverse bacterial species.****

| **Integron (n)** | ***bla*_IMP_ (species, n)** |
| --- | --- |
| -^a^ (2) | *bla*_IMP-27_ (*Providencia stuartii*, 2) |
| 2 (2) | *bla*_IMP-27_ (*Morganella morganii*^b^, 1; *Proteus mirabilis*^b^, 1) |
| 5 (1) | *bla*_IMP-79_ (*Pseudomonas aeruginosa*, 1) |
| 14 (2) | *bla*_IMP-87_ (*Pseudomonas aeruginosa*, 1); *bla*_IMP-91_ (*Pseudomonas aeruginosa*, 1) |
| 45 (4) | *bla*_IMP-6_ (*Pseudomonas aeruginosa*, 1); *bla*_IMP-18_ (*Pseudomonas aeruginosa*, 2); *bla*_IMP-75_ (*Pseudomonas aeruginosa*, 1) |
| 49 (2) | *bla*_IMP-1_ (*Pseudomonas aeruginosa*, 1); *bla*_IMP-8_ (*Enterobacter cloacae*, 1) |
| 240 (2) | *bla*_IMP-4_ (*Klebsiella pneumoniae*^c^, 1); *bla*_IMP-19_ (*Acinetobacter baumannii*, 1) |
| 336 (2) | *bla*_IMP-1_ (*Pseudomonas juntendi*, 1); *bla*_IMP-8_ (*Alcaligenes faecalis*, 1) |
| 449 (1) | *bla*_IMP-13_ (*Pseudomonas aeruginosa*, 1) |
| 498 (25) | *bla*_IMP-1_ (*Pseudomonas putida*, 2); *bla*_IMP-4_ (*Klebsiella pneumoniae*^c^, 1); *bla*_IMP-6_ (*Pseudomonas aeruginosa*^c^, 3); *bla*_IMP-8_ (*Aeromonas caviae*, 1; *Comamonas aquatica*, 1); *bla*_IMP-13_ (*Pseudomonas aeruginosa*, 1); *bla*_IMP-15_ (*Pseudomonas juntendi*, 1); *bla*_IMP-18_ (*Pseudomonas aeruginosa*, 3); *bla*_IMP-45_ (*Pseudomonas aeruginosa*, 1); *bla*_IMP-48_ (*Achromobacter xylosoxidans*, 2; *Pseudomonas putida*, 1); *bla*_IMP-79_ (*Pseudomonas putida*, 1; *Pseudomonas aeruginosa*, 6); *bla*_IMP-84_ (*Pseudomonas aeruginosa*, 1) |
| 786 (1) | *bla*_IMP-45_ (*Pseudomonas aeruginosa*, 1) |
| 871 (1) | *bla*_IMP-1_ (*Pseudomonas* sp., 1) |
| 887 (4) | *bla*_IMP-6_ (*Pseudomonas aeruginosa*^c,d^, 4) |
| 990 (2) | *bla*_IMP-15_ (*Pseudomonas aeruginosa*, 2) |
| 994 (2) | *bla*_IMP-1_ (*Pseudomonas aeruginosa*^e^, 1); *bla*_IMP-79_ (*Pseudomonas aeruginosa*, 1) |
| 1060 (1) | *bla*_IMP-8_ (*Comamonas thiooxydans*, 1) |
| 1065 (1) | *bla*_IMP-96_ (*Stenotrophomonas* sp., 1) |
| 1069 (1) | *bla*_IMP-15_ (*Pseudomonas aeruginosa*, 1) |
| 1114 (1) | *bla*_IMP-79_ (*Pseudomonas aeruginosa*, 1) |
| 1469 (2) | *bla*_IMP-13_ (*Enterobacter hormaechei*, 1; *Pseudomonas aeruginosa*, 1) |

^a^The *bla*_IMP_ gene was positioned in close proximity to or in the integron.

^b^The *bla*_IMP_ gene was positioned in close proximity to the integron but not directly within the integron.

^c^The strain harbored two different *bla*_IMP_-integrons.

^d^One strain harbored a duplicated *bla*_IMP_-integron.

^e^The strain harbored a duplicated *bla*_IMP_-integron.

*Pseudomonas putida* *bla*_IMP-19_ gene was used as the reference sequence to identify strains with chromosomal-integrated *bla*_IMP_.

BacAnt (1) was used to identify integrons and their composition.

**Table S5. *bla*_IMP_ genes identified in 56 bacterial chromosomes.**

| **Strain** | **Accession** | **Most like integron** | **Identity/coverage (%)** | **Antimicrobial resistance genes** |
| --- | --- | --- | --- | --- |
| *Acinetobacter baumannii* AB322 | CP119232 | In240|EF368053 | 94.78/89.66 | *aac(6')-Ib4*, *bla*_IMP-19_ |
| *Alcaligenes faecalis* NY11312 | CP096916 | In336|GQ422829 | 98.71/19.89 | *bla*_IMP-8_ |
| *Aeromonas caviae* NY4617 | CP047981 | In498|AY214164 | 99.8/100.0 | *bla*_IMP-8_ |
| *Achromobacter xylosoxidans* R8 | LN890477 | In498|AY214164 | 99.9/100.0 | *bla*_IMP-48_ |
| *Achromobacter xylosoxidans* R4 | LN890476 | In498|AY214164 | 99.9/100.0 | *bla*_IMP-48_ |
| *Comamonas aquatica* NY8661 | CP096918 | In498|AY214164 | 99.9/100.0 | *bla*_IMP-8_ |
| *Comamonas thiooxydans* ZDHYF418 | CP063057 | In1060|KF468742 | 99.89/40.86 | *bla*_IMP-8_ |
| *Enterobacter cloacae* A1137 | CP021851 | In49|EU434618 | 99.31/79.23 | *aac(6')-Ib4*, *bla*_IMP-8_ |
| *Enterobacter hormaechei* 2020CK-00224 | CP118383 | In1469|LC333379 | 100.0/40.06 | *bla*_IMP-13_ |
| *Klebsiella pneumoniae* CPO109^c^ | CP117837 | In240|EF368053  In498|AY214164 | 97.25/23.43  99.8/100.0 | *bla*_IMP-4_, *catB3*  *bla*_IMP-4_, *catB3*, *emrE* |
| *Morganella morganii* N18-00103 | CP048275 | In2-45|FJ474094 | 98.81/93.02 | *bla*_IMP-27_^a^ |
| *Proteus mirabilis* N18-00201 | CP048404 | In2-12|EU732664 | 99.1/42.25 | *bla*_IMP-27_^a^ |
| *Providencia stuartii* 2021CK-01196 | CP114580 | - | - | *bla*_IMP-27_^b^ |
| *Providencia stuartii* 2021CK-01296 | CP114582 | - | - | *bla*_IMP-27_^b^ |
| *Stenotrophomonas* sp. NY11291 | CP096975 | In1065|KF468748 | 94.78/97.94 | *bla*_IMP-96_ |
| *Pseudomonas sp.* NY5710 | CP045554 | In871|HQ662555 | 99.81/22.33 | *aadA1*, *bla*_OXA-21_, *bla*_IMP-1_ |
| *Pseudomonas juntendi* 18091276 | CP091311 | In336|GQ422829 | 100.0/19.06 | *bla*_IMP-1_ |
| *Pseudomonas juntendi* PP_2463 | CP091088 | In498|AY214164 | 100.0/100.0 | *bla*_IMP-15_ |
| *Pseudomonas putida* MRSN365855 | CP132007 | In498|AY214164 | 100.0/100.0 | *emrE*, *bla*_IMP-48_ |
| *Pseudomonas putida* NY5709 | CP045551 | In498|AY214164 | 99.9/100.0 | *emrE*, *bla*_OXA-21_, *bla*_IMP-1_ |
| *Pseudomonas putida* AHSWHJXPP1 | CP120969 | In498|AY214164 | 100.0/100.0 | *aac(6')-IIa*, *bla*_IMP-1_ |
| *Pseudomonas putida* NY4811 | CP096920 | In498|AY214164 | 99.9/100.0 | *bla*_IMP-79_ |
| *Pseudomonas aeruginosa* 401853 | MZ702721 | - | - | *bla*_IMP-91_ |
| *Pseudomonas aeruginosa* NY5511 | CP096934 | In5|PAU38230 | 100.0/7.8 | *bla*_IMP-79_ |
| *Pseudomonas aeruginosa* KB-PA_3 | CP086016 | In14|AM412777 | 98.17/90.99 | *bla*_OXA-10_, *bla*_IMP-87_ |
| *Pseudomonas aeruginosa* NY3045 | CP059995 | In14|AM412777 | 98.17/90.99 | *bla*_OXA-10_, *bla*_IMP-91_ |
| *Pseudomonas aeruginosa* HPA0118 | CP137522 | In45|FJ460240 | 93.89/93.53 | *aadA1*, *bla*_OXA-1_, *bla*_IMP-6_ |
| *Pseudomonas aeruginosa* 2021CK-01104 | CP137913 | In45|FJ460240 | 93.28/93.1 | *aadA1*, *bla*_IMP-18_ |
| *Pseudomonas aeruginosa* 2021CK-01161 | CP124626 | In45|FJ460240 | 93.28/93.1 | *aadA1*, *bla*_IMP-18_ |
| *Pseudomonas aeruginosa* 2022CK-00339 | CP124674 | In45|FJ460240 | 93.28/93.1 | *aadA1*, *bla*_IMP-75_ |
| *Pseudomonas aeruginosa* CUVET20-956 | CP116934 | In49|EU434618 | 99.39/88.8 | *bla*_IMP-1_ |
| *Pseudomonas aeruginosa* 3541 | OX638610 | In449|JN091097 | 99.66/96.68 | *bla*_IMP-13_ |
| *Pseudomonas aeruginosa* IMP-13 | CP034354 | In498|AY214164 | 99.9/100.0 | *bla*_IMP-13_ |
| *Pseudomonas aeruginosa* HB2011305RE | CP054787 | In498|AY214164 | 99.9/100.0 | *bla*_IMP-79_ |
| *Pseudomonas aeruginosa* P93127 | CP087675 | In498|AY214164 | 100.0/100.0 | *bla*_OXA-10_, *bla*_IMP-79_ |
| *Pseudomonas aeruginosa* 3796A | OX638564 | In498|AY214164 | 99.9/100.0 | *bla*_IMP-84_ |
| *Pseudomonas aeruginosa* AG1 | CP045739 | In498|AY214164 | 99.9/100.0 | *bla*_OXA-2_, *bla*_IMP-18_ |
| *Pseudomonas aeruginosa* 2021CK-01162 | CP124632 | In498|AY214164 | 99.8/100.0 | *aadA1*, *bla*_IMP-18_ |
| *Pseudomonas aeruginosa* 2020CK-00218 | CP124649 | In498|AY214164 | 99.8/100.0 | *aadA1*, *bla*_IMP-18_ |
| *Pseudomonas aeruginosa* 97 | CP031449 | In498|AY214164 | 100.0/100.0 | *bla*_OXA-10_, *bla*_IMP-79_ |
| *Pseudomonas aeruginosa* 12939 | CP024477 | In498|AY214164 | 99.9/100.0 | *bla*_IMP-79_ |
| *Pseudomonas aeruginosa* NY7483 | CP120858 | In498|AY214164 | 100.0/100.0 | *bla*_IMP-79_ |
| *Pseudomonas aeruginosa* HPA0044^c^ | CP137505 | In498|AY214164  In887|KC960557 | 99.9/100.0  99.97/94.67 | *aadA1*, *bla*_OXA-1_, *bla*_IMP-6_  *aadA1*, *bla*_OXA-1_, *bla*_IMP-6_ |
| *Pseudomonas aeruginosa* HPA0384^c^ | CP137500 | In498|AY214164  In887|KC960557 | 99.9/100.0  99.97/94.67 | *aadA1*, *bla*_OXA-1_, *bla*_IMP-6_  *aadA1*, *bla*_OXA-1_, *bla*_IMP-6_ |
| *Pseudomonas aeruginosa* HPA1406^c^ | CP137491 | In498|AY214164  In887|KC960557 | 99.9/100.0  99.88/94.67 | *aadA1*, *bla*_OXA-1_, *bla*_IMP-6_  *aadA1*, *bla*_OXA-1_, *bla*_IMP-6_ |
| *Pseudomonas aeruginosa* P4970C | CP087674 | In498|AY214164 | 100.0/100.0 | *bla*_OXA-10_, *bla*_IMP-79_ |
| *Pseudomonas aeruginosa* NY11084 | CP120705 | In498|AY214164 | 99.8/100.0 | *bla*_OXA-1_, *bla*_IMP-45_ |
| *Pseudomonas aeruginosa* 59 | CP123953 | In786|EU588392 | 99.98/76.7 | *bla*_OXA-1_, *bla*_IMP-45_ |
| *Pseudomonas aeruginosa* HPA0875^d^ | CP137495 | In887|KC960557 | 99.88/94.67 | *aadA1*, *bla*_OXA-1_, *bla*_IMP-6_ |
| *Pseudomonas aeruginosa* ZBX-P12 | CP061779 | In990|KP177455 | 92.11/92.99 | *aadA1*, *bla*_IMP-15_ |
| *Pseudomonas aeruginosa* ZBX-P23 | CP061777 | In990|KP177455 | 92.11/92.99 | *aadA1*, *bla*_IMP-15_ |
| *Pseudomonas aeruginosa* PA99^d^ | CP042967 | In994|AB901045 | 100.0/98.77 | *bla*_IMP-1_ |
| *Pseudomonas aeruginosa* PALA20 | CP107064 | In994|AB901045 | 99.48/98.77 | *bla*_IMP-79_ |
| *Pseudomonas aeruginosa* 2020CK-00185 | CP124646 | In1069|KM589497 | 96.03/35.85 | *bla*_OXA-2_, *bla*_IMP-15_, *catB2* |
| *Pseudomonas aeruginosa* NY7770 | CP096912 | In1114|KF914309 | 99.63/85.23 | *aadA1*, *bla*_IMP-79_ |
| *Pseudomonas aeruginosa* 2858 | CP116718 | In1469|LC333379 | 100.0/40.06 | *emrE*, *catB3*, *bla*_OXA-1_, *aac(6')-Ib-cr*, *aac(6')-II*, *bla*_OXA-2_, *bla*_IMP-13_ |

^a^The *bla*_IMP_ gene was positioned in close proximity to the integron but not directly within the integron.

^b^The *bla*_IMP_ gene was positioned in close proximity to or in the integron.

^c^The strain harbored two different *bla*_IMP_-integrons.

^d^The strain harbored a duplicated *bla*_IMP_-integron.

*Pseudomonas putida* *bla*_IMP-19_ gene was used as the reference sequence to identify strains with chromosomal-integrated *bla*_IMP_.

BacAnt (1) was used to identify integrons and their composition.

**Table S6. The presence of phages on the AB322 chromosome.**

| **Region** | **Region length (Kb)** | **Completeness** | **Score** | **Total proteins (n)** | **Region position** | **Most common phage** | **GC (%)** |
| --- | --- | --- | --- | --- | --- | --- | --- |
| 1 | 74.3 | intact | 110 | 84 | 419661-494049 | PHAGE_Acinet_YMC11/11/R3177_NC_041866(36) | 38.68 |
| 2 | 17.8 | incomplete | 60 | 20 | 689419-707218 | PHAGE_Psychr_pOW20_A_NC_020841(5) | 37.24 |
| 3 | 40.1 | incomplete | 50 | 36 | 1134354-1174511 | PHAGE_Bordet_BPP_1_NC_005357(12) | 41.21 |
| 4 | 33.6 | questionable | 90 | 44 | 1937106-1970709 | PHAGE_Pelagi_HTVC010P_NC_020481(4) | 40.97 |
| 5 | 47.1 | intact | 120 | 73 | 2025605-2072739 | PHAGE_Acinet_Bphi_B1251_NC_019541(33) | 38.36 |
| 6 | 75.1 | intact | 140 | 87 | 2286451-2361572 | PHAGE_Acinet_vB_AbaS_TRS1_NC_031098(13) | 39.70 |
| 7 | 38.7 | incomplete | 30 | 41 | 2968528-3007278 | PHAGE_Salmon_SJ46_NC_031129(3) | 55.36 |
| 8 | 31.2 | intact | 150 | 49 | 3780995-3812236 | PHAGE_Pseudo_Dobby_NC_048109(16) | 39.12 |
| 9 | 53.8 | intact | 150 | 83 | 3967282-4021093 | PHAGE_Acinet_YMC11/11/R3177_NC_041866(32) | 39.58 |

The PHASTER server (2) was employed to identify phages.

Intact (score > 90); Questionable (score 70-90); Incomplete (score < 70).

**Fig. S1**


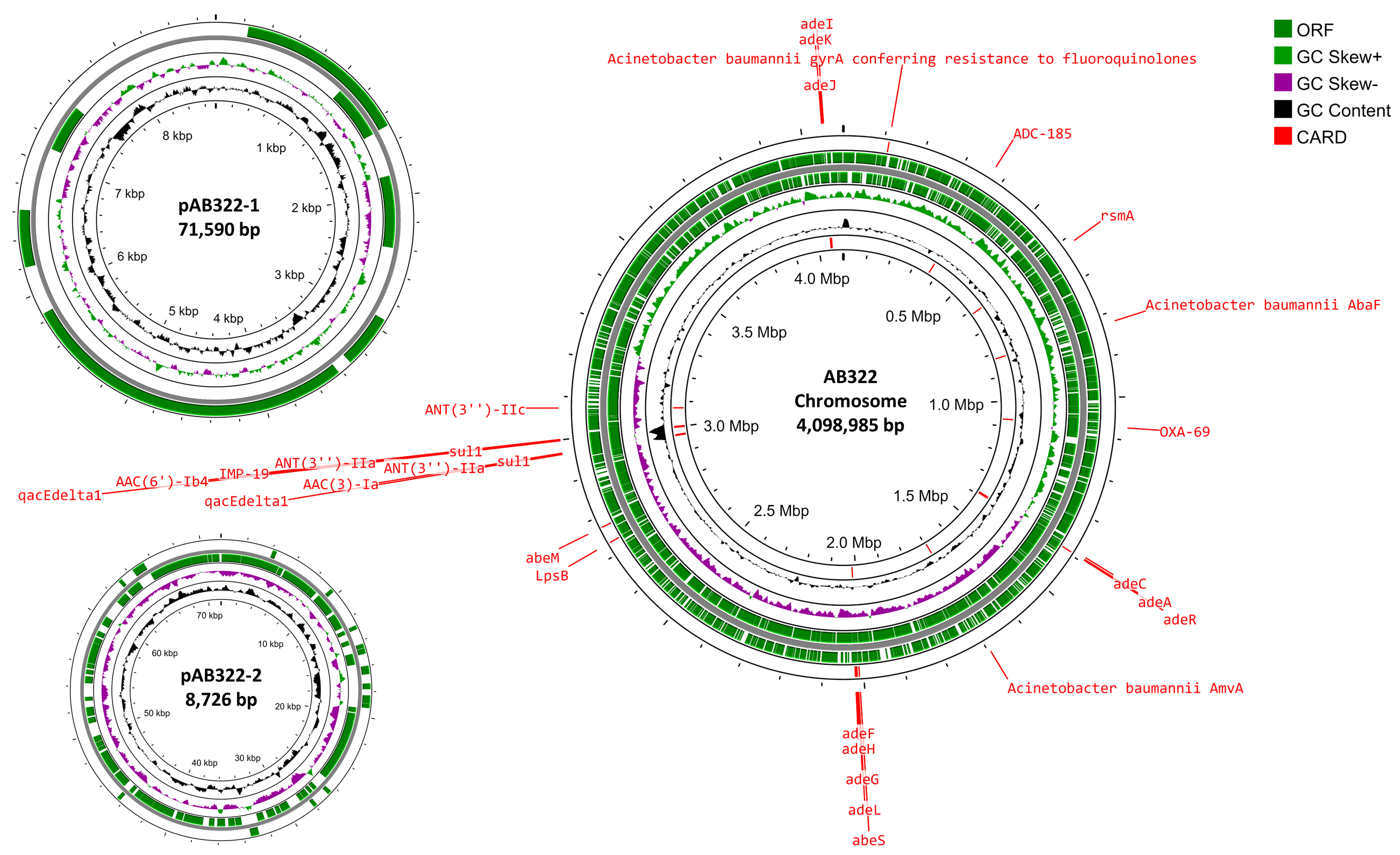


**Fig. S1. Circular genome map of A. baumannii AB322 generated using the GCView server.** The scales indicate the location in Mbp (chromosome) or Kbp (plasmids), commencing from the initial coding region. Starting from the innermost circles, circle 1 illustrates the GC content, which is depicted using a sliding window. Circle 2 represents the GC skew (G-C/G+C), with values plotted as the deviation from the average GC skew of the entire sequence. Circles 3 and 4 show the coding sequences; circle 3 represents the backward strand, and circle 4 represents the forward strand. The prediction of antimicrobial resistance genes in the chromosome was carried out using the Comprehensive Antibiotic Resistance Database (CARD) and presented in red labeling. No antimicrobial resistance genes were identified in pAB322-1 and pAB322-2.

**Fig. S2.**





**Fig. S2. Prophages distribution on AB322 chromosome.** The PHASTER analysis identified the locations of 9 prophages on the AB322 chromosome. Green indicates intact prophages (score >90), blue represents questionable prophages (score 70-90), and red indicates incomplete prophages (score <70). The *bla*_IMP-19_ gene is located in region 7.

**References:**

1. Hua X, Liang Q, Deng M, He J, Wang M, Hong W, Wu J, Lu B, Leptihn S, Yu Y, Chen H. 2021. BacAnt: A Combination Annotation Server for Bacterial DNA Sequences to Identify Antibiotic Resistance Genes, Integrons, and Transposable Elements. Front Microbiol 12:649969.

2. Arndt D, Grant JR, Marcu A, Sajed T, Pon A, Liang Y, Wishart DS. 2016. PHASTER: a better, faster version of the PHAST phage search tool. Nucleic Acids Res 44:W16-21.
